# Hepatitis C virus E1 and modified E2 delivered from an mRNA vaccine induces protective immunity

**DOI:** 10.1038/s41541-023-00635-9

**Published:** 2023-03-18

**Authors:** Tapas Patra, Keith Meyer, Yuki Haga, Erin K. Reagan, Drew Weissman, Ranjit Ray

**Affiliations:** 1 Department of Internal Medicine, Saint Louis University, Missouri, MO 63104 USA; 2 Department of Medicine, University of Pennsylvania, Philadelphia, PA 19104 USA; 3 Department of Molecular Microbiology & Immunology, Saint Louis University, Missouri, MO 63104 USA

**Keywords:** Infectious diseases, RNA vaccines

## Abstract

Hepatitis C virus (HCV) is characterized by a high number of chronic cases due to an impairment of protective innate and adaptive immune responses. Here, we examined the contribution of the individual ectodomains of E1, E2, or a modified E2 with reduced CD81 binding and an inserted N-linked glycosylation site in combination as vaccine antigen mRNA-lipid nanoparticles (LNPs). The induction of a protective immune response to surrogate recombinant vaccinia virus (VV) expressing homologous HCV glycoprotein(s) challenge infection in a BALB/c mouse model was observed. Vaccination with a mRNA-LNP expressing soluble E1 (sE1) significantly reduced vv/HCV titer in the mouse ovary. However, the addition of sE2 mRNA-LNP for immunization impaired the efficacy of the sE1 construct. Further analysis showed that Th1 related cytokine responses to the sE1 mRNA-LNP were significantly altered in the presence of sE2 following co-immunization. Evaluation of immunogenicity revealed that the use of modified sE2_F442NYT_ nucleoside mRNA-LNP vaccine results in an improved cellular immune response, IgG2a isotype switching, enhanced total IgG, and an increase in the neutralizing antibody response against HCV pseudotype virus. HCV cross genotype specific reactivity to peptides representing conserved E2 specific linear epitopes were enhanced in modified E2 vaccinated animal sera. In the absence of a suitable immunocompetent small animal model for HCV infection, protection from surrogate HCV vaccinia challenge infection model was observed in the immunized mice as compared to sE1 alone or an unmodified sE2 mRNA-LNP vaccine. Inclusion of sE1 with modified sE2_F442NYT_ as mRNA-LNP vaccine candidate appeared to be beneficial for protection.

## Introduction

Hepatitis C virus (HCV) infection causes silent liver disease and is a major health problem worldwide. A comprehensive strategy to control HCV infection must include an effective vaccine development approach. Obstacles to vaccine development in the HCV field include a limited ability to test in an animal model, the diverse nature of HCV genotypes and subtypes, what constitutes a protective immune response, and which viral antigen would best achieve protection against infection. The HCV E1 envelope glycoprotein is relatively conserved and neutralizing antibodies were developed in response to vaccination with recombinant E1 glycoprotein in chimpanzees^1^. We and others have characterized linear epitopes from HCV E1^2,3^. In a phase I study, a candidate E1 therapeutic vaccine delivered as virus-like particles was well tolerated and immunogenic in healthy, adult males^4^. Additionally, a cellular immune response toward E1 was elicited, which included a strong Th1 component. Our study suggested that HCV E2 possesses immunoregulatory activities, which may be a reason for the impairment of a strong immune response to E1 when used in combination^5–7^.

In an earlier study, we used purified HCV E1/E2 envelope glycoproteins in a phase 1 vaccine trial in humans and observed the induction of a Th2 biased, weak immune response^5,6^. MF59 was used as an adjuvant in the vaccine trial to aid in the induction of a Th1 cytokine profile, CD4^+^T memory cells, and cytotoxic T lymphocytes. However, the effect of MF59 appeared to be reduced in the immunized volunteers, as suggested from IL-10 induction, and an undetectable level of IL-12. A similar induction of IL-10, but not IL-12, was observed using human macrophages in our subsequent in vitro study using purified recombinant E2 glycoprotein^7^, and reinforced in a recent study using in vitro samples, and vaccinated mice^8^. HCV entry into hepatocytes is facilitated by binding with CD81, which represents a major site of vulnerability for consideration in vaccine design. We examined whether impairing the E2-CD81 interaction by using modified E2 with point mutations and the insertion of a glycosylation site (F442NYT) altered cytokine parameters associated with an improved proinflammatory response. Soluble ectodomain regions of E1/E2_F442NYT_ delivered as an mRNA-LNP candidate vaccine exhibited improved T-helper cell function, neutralizing antibody response, and afforded robust cellular immunity in mice during a surrogate challenge infection using recombinant vaccinia virus expressing HCV E1-E2-NS2 (aa134–966)^8^. Our findings could potentially lead to the further development of a multi-genotype mRNA- LNP HCV vaccine approach to promote protective humoral and cellular immune responses for future human use.

HCV E2 epitopes, including the highly conserved amino acid residues 412–423 of E2, cover areas which are critical for CD81 receptor binding^9–11^. These may be targeted by antibodies isolated from human and animal sera. An examination of the antigenic role of HCV E2 by human antigen-presenting cells; and their incorporation into a candidate vaccine, may be balanced by the ability of E2 to modulate immune cell function. Our mechanistically guided approach will likely enable the use of selected conserved antigenic targets in a candidate vaccine which are cross-reactive among HCV genotypes. Thus, careful selection of E2-associated immunogens, and other relatively conserved appropriate antigen(s) (including E1 with or without the non-structural regions), will significantly contribute to the development of a highly effective HCV vaccine.

HCV envelope glycoproteins, E1 and E2 contain multiple epitopes for both T and B cells^2,12–15^. We recently examined whether impairing the E2-CD81 interaction can promote stronger T-helper cell function and induce a robust HCV antigen-specific immune responses^8^. Our previous results indicated that immunization with native HCV E2 has reduced cellular immunity, a decreased ability to neutralize surrogate HCVpp from the same genotype (H77), and has a cytokine profile that indicates a reduced potential for a Th1 response. Here, we examined the nature of immunogenicity of the E1 protein with or without sE2 mRNA-LNP vaccine in mice. Further, we have used a surrogate recombinant vaccinia virus (vv)/HCV challenge model to test the biological relevance of the T cell response as a function for protection from infection. Interestingly, our results suggested that wild type E2 inhibited the E1 specific cellular immune response, and suppressed the E1 specific cytokine response; as compared to animals vaccinated with E1 alone. These results further suggested an immunoregulatory role for the unmodified E2 glycoprotein of HCV that extended to the E1 specific response in a candidate envelope glycoprotein vaccine development approach.

## Results

### A modified HCV envelope mRNA-LNP vaccine confers protective cellular immunity

**Fig. 1:**
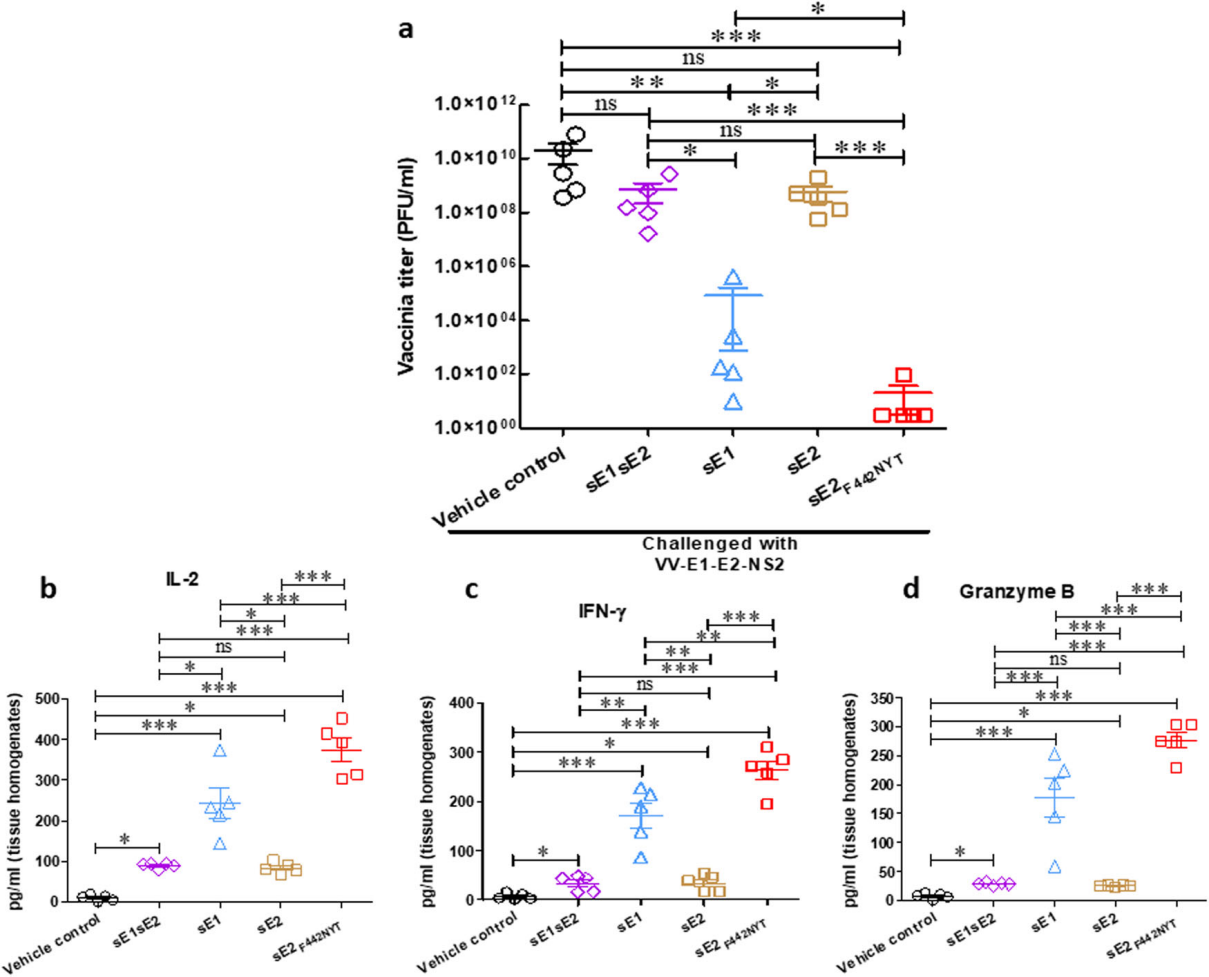
Protective responses of HCV sE1/sE2, sE1, sE2 or sE2_F442NYT_ mRNA-LNP immunized mice. **a** Recombinant vaccinia virus titers were determined by plaque forming assay from the ovaries of immunized BALB/c mice after the vaccinia virus (expressing HCV E1-E2-NS2_aa134–966_) challenge. **b**–**d** The levels of IL-2, IFN-γ and granzyme B were quantified by ELISA from ovary homogenates of the recombinant vaccinia challenged mice. The results are presented as the mean with SD. ‘*’, ‘**’, ‘***’ considered statistical significance (ANOVA and t-test) with *p*-value of < 0.05, < 0.005, < 0.0005, and ‘ns’ denotes as non-significant.

The absence of an appropriate small animal model which mimics the natural host for HCV infection is one of the biggest obstacles in evaluating vaccine efficacy. To assess the biological relevance of a protective cellular immune response induced by mRNA-LNP vaccination with different HCV envelope constructs, we utilized a surrogate challenge model of a recombinant vaccinia virus expressing HCV E1-E2-NS2. The recombinant virus was intraperitoneally inoculated (10^7^ pfu in 200 µl) 2 weeks after the second mRNA-LNP vaccination of BALB/c mice. The mice were sacrificed 4 days later, and the ovaries were harvested for vaccinia virus recovery. We observed that mice immunized with sE1/sE2 mRNA-LNP in combination or sE2 mRNA-LNP alone demonstrated ~1-log reduction (*p* = 0.1348 and *p* = 0.1223; one way ANOVA test) in the mean virus titer in comparison to the mRNA-LNP vehicle control group (Fig. 1a). However, four of the five mice immunized with the modified sE2_F442NYT_ did not exhibit a detectable virus titer after challenge infection with recombinant vaccinia virus, while the remaining animal had an ~8-log reduction in virus titer (*p* = 0.0006; one way ANOVA test). Interestingly, the sE1 alone immunized mouse group also had a significant decrease in viral titer (>4 log, *p* = 0.0097; one way ANOVA test) as compared to the control group (Fig. 1a). Thus, the modified sE2 _F442NYT_ mRNA-LNP vaccine preparation induced the strongest protective immune response in our surrogate challenge animals. Our results further exhibited that the protective cellular response derived from vaccination with mRNA-LNPs expressing HCV E1 was greatly reduced in the presence of native HCV E2 mRNA-LNPs, indicating that HCV E2 may have a modulating effect upon co-inoculated immunogens.

Earlier studies suggested that a surrogate recombinant vaccinia virus challenge model confers tissue specific cellular immunity. We analyzed the tissue specific level of IL-2 and IFN-γ in the clarified ovary homogenate of the recombinant vaccinia virus challenged mice used in the above experiments, and observed a significant increase in both cytokines in mice immunized with the modified sE2 _F442NYT_ mRNA-LNP vaccine (Fig. 1b–d). The sE1 alone immunized group also showed a slightly less substantial increase in expression of IL-2 and IFN-γ. However, sE1/sE2 in combination or sE2 alone immunized mice exhibited a very low expression level of these cytokines.

Granzyme B is a key cytolytic mediator released from cytotoxic T lymphocytes (CTL) into virus-infected tissues which helps in clearing viral infections^16^. Tissue specific levels of granzyme B are a traditional determinant of CTL mediated activity. Several studies have measured tissue specific granzyme B level along with other cytokines as an indicator of cellular immunity^17^. Granzyme B expression in the ovary homogenates of sE2_F442NYT_ immunized mice was increased as was; to a lesser extent, Granzyme B in sE1 immunized mice (Fig. 1b–d). These results correlated with detectable vaccinia titer reduction, indicating that CTL-mediated immunity was generated in this surrogate mouse model. Further, Granzyme B expression in sE1/sE2-immunized mice was significantly lower than expression of sE1 immunized mice, suggesting that sE2 may act to suppress cytokine and cellular immune responses generated by co-expressed molecules.

### Modified sE2 mRNA-LNP elevates neutralizing antibody response

**Fig. 2:**
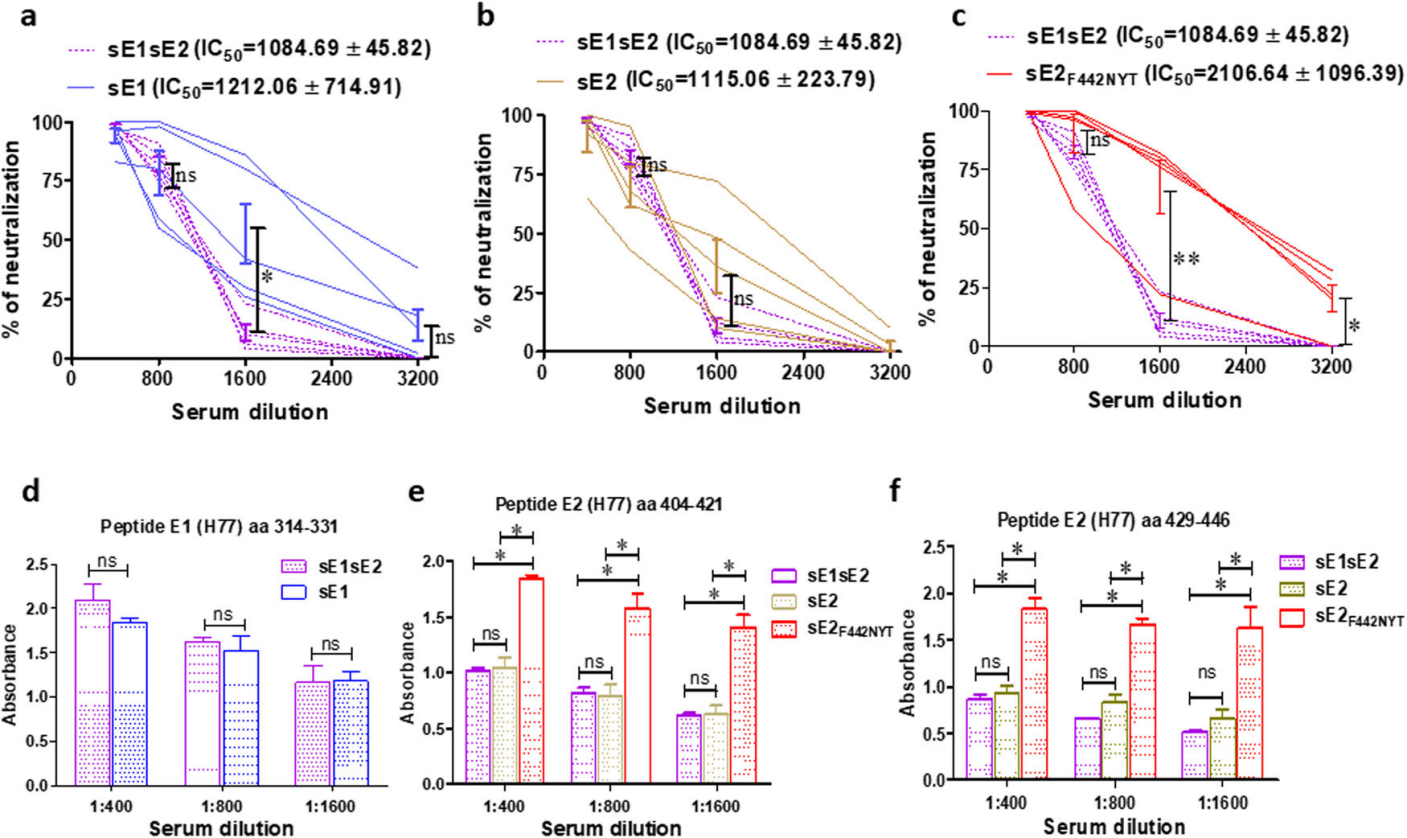
Antibody responses of HCV sE1/sE2, sE1, sE2 or sE2_F442NYT_ mRNA-LNP immunized mice. **a**–**c** Comparative neutralization of lentivirus derived HCVpp from genotype 1a by sera from immunized mice at different dilutions. The IC50 value of each vaccinated group is indicated as the exact dilution of the serum showing 50% neutralizing activity in each vaccinated group of 5 mice with mean ± SD. **d–f** Serum antibody reactivity from immunized mice to HCV E1 (aa 314–331) and E2 (aa 404–421 and aa 429–446) specific peptides were determined. The results are presented as the mean with SD. ‘*’, ‘**’ considered statistical significance (Mann-Whitney U and t-test) with *p*-value of < 0.05, < 0.005 and ‘ns’ denotes as non-significant.

Neutralizing antibody response to antigens corresponds to a functional immune response, and often contributes to protection from virus infection. To understand the comparative antibody neutralization of each HCV envelope-mRNA candidate vaccine, we analyzed HCV-lentiviral pseudotype particles (HCVpp) representing homologous H77C neutralization by serum from individual vaccinated mice. The sE2_F442NYT_ immunized group displayed an almost 2-fold greater reduction of HCVpp luciferase activity, implying enhanced neutralization efficacy, as compared to the sE1/sE2 combined immunization (IC_50_ = 2106.64 ± 1096.39 vs. IC_50_ = 1084.69 ± 45.82) (mean ± SD). Vaccination with solitary sE2 did not reflect any significant difference from the combined sE1/sE2 immunization (IC_50_ = 1115.06 ± 223.79 vs. 1084.69 ± 45.82) (mean ± SD) (Fig. 2a–c). In some cases, sera from sE1 immunized mice exhibited higher neutralization (IC_50_ = 1212.06 ± 714.91) (mean ± SD) in comparison to the combined sE1/sE2 immunized group, but this was not prevalent in all immunized animals.

The ability to cross neutralize different genotypes of HCV may be an important feature of vaccine efficacy. We extended our effort in preparing HCV lentiviral psuedotypes from genotypes 1b (1b58), 3 (3.1.2), and 4 (4.1.2) using plasmid DNAs. Cross genotype neutralization analyses of pseudotypes by a pool of HCV vaccinated sera stored from our previous phase I vaccine trial using the envelope glycoproteins E1/E2 of HCV (H77) with neutralization activity to both HCVpp and cell culture grown HCV^5,6^ were used for the initial test. Neutralization of pseudoparticles (Table 1) suggested a titer of 1:400 against HCVpp from genotype 1a, with a 2-fold reduction in neutralization using HCVpp from genotype 4 (1:200), and a 4-fold reduction in neutralization of the HCVpp genotype 1b and 3 (1:100). The observations agree with those reported earlier, showing that the vaccine sera had a reduced level of cross genotype neutralization^18^. Subsequently, we analyzed HCV-mRNA-LNP vaccinated pooled mouse sera for HCVpp cross genotype neutralization (Table 2). Mice vaccinated with sE2 or sE2_F442NYT_ mRNA-LNP with modified E2 (H77) showed an increased neutralization. A 2-fold decrease in neutralization titer with HCVpp from genotypes 1b, 3 and 4 (1:200) as compared to the genotype 1a of the vaccine strain (1:400) using sE2 vaccinated pooled mouse sera was observed. In contrast, the pooled mouse sera from sE2_F442NYT_ vaccinated mice exhibited an increase in neutralization titers for 1a (1:1600), 1b (1:1600) and 4 (1:400). The neutralization titer for genotype 3 was not higher in sE2_F442NYT_ vaccinated pooled mouse sera than that seen with sE2 vaccinated sera. While the overall results are very optimistic for enhanced cross genotype neutralization with the sE2_F442NYT_ mRNA-LNP, the information using HCV genotype 3 indicate that the use of multiple modified HCV E2 sequences may be necessary to acquire a vaccine with pan-genotypic efficiency of neutralizing antibody.

**Table 1:** HCVpp genotype specific neutralization by pooled HCV1a envelope glycoproteins immunized human sera.

Reciprocal dilution	Genotype1a	Genotype1b	Genotype 3	Genotype 4
100	89 ± 8	63 ± 1	72 ± 12	70 ± 0.5
200	69 ± 2	< 50	< 50	68 ± 8
400	58 ± 5	< 50	< 50	< 50
800	< 50	< 50	< 50	< 50

**Table 2:** HCVpp genotype specific neutralization by pooled immunized mouse sera.

Reciprocal dilution	Genotype1a	Genotype1b	Genotype 3	Genotype 4	Genotype1a	Genotype1b	Genotype 3	Genotype 4
200	82 ± 4	69 ± 2	93 ± 4	88 ± 6	97 ± 0.3	98 ± 0.4	88 ± 2	84 ± 2
400	74 ± 3	< 50	< 50	< 50	96 ± 1	92 ± 4	< 50	92 ± 3
800	< 50	< 50	< 50	< 50	96 ± 1	97 ± 1	< 50	< 50
1600	< 50	< 50	< 50	< 50	63 ± 7	94 ± 3	< 50	< 50
	sE2-mRNA-LNP immunized				sE2_F442NYT-_mRNA-LNP immunized			

We extended our study to investigate whether the modified HCV envelope-mRNA candidate vaccine induces antibodies particularly recognizing the conserved E1 or E2 epitope associated with broad neutralization activity. For this, we used one E1 based peptide spanning the amino acid region 314–331, and two E2 based peptides spanning amino acids 404–421 and 429–446, which are responsible for HCV multi-genotype neutralization^9,10,12–15^. ELISA was performed by coating these peptides and using serially diluted vaccinated mouse sera. Analyses involving ELISA reactivity using specific peptides identified minimal changes in reactivity to a conserved E1 specific peptide. In contrast, immunization of mice with the sE2_F442NYT_ construct conveyed a significant increase in reactivity to both E2 specific peptides at dilutions shown here (Fig. 2d–f). Thus, our results suggested that immunization with the modified sE2_F442NYT_ mRNA-LNP vaccine elicits a stronger antibody binding response (4-fold higher), as compared to the unmodified envelope glycoproteins.

### Th1 specific cytokine and immunoglobulin subclass responses in sE2_F442NYT_ vaccinated mouse sera

**Fig. 3:**
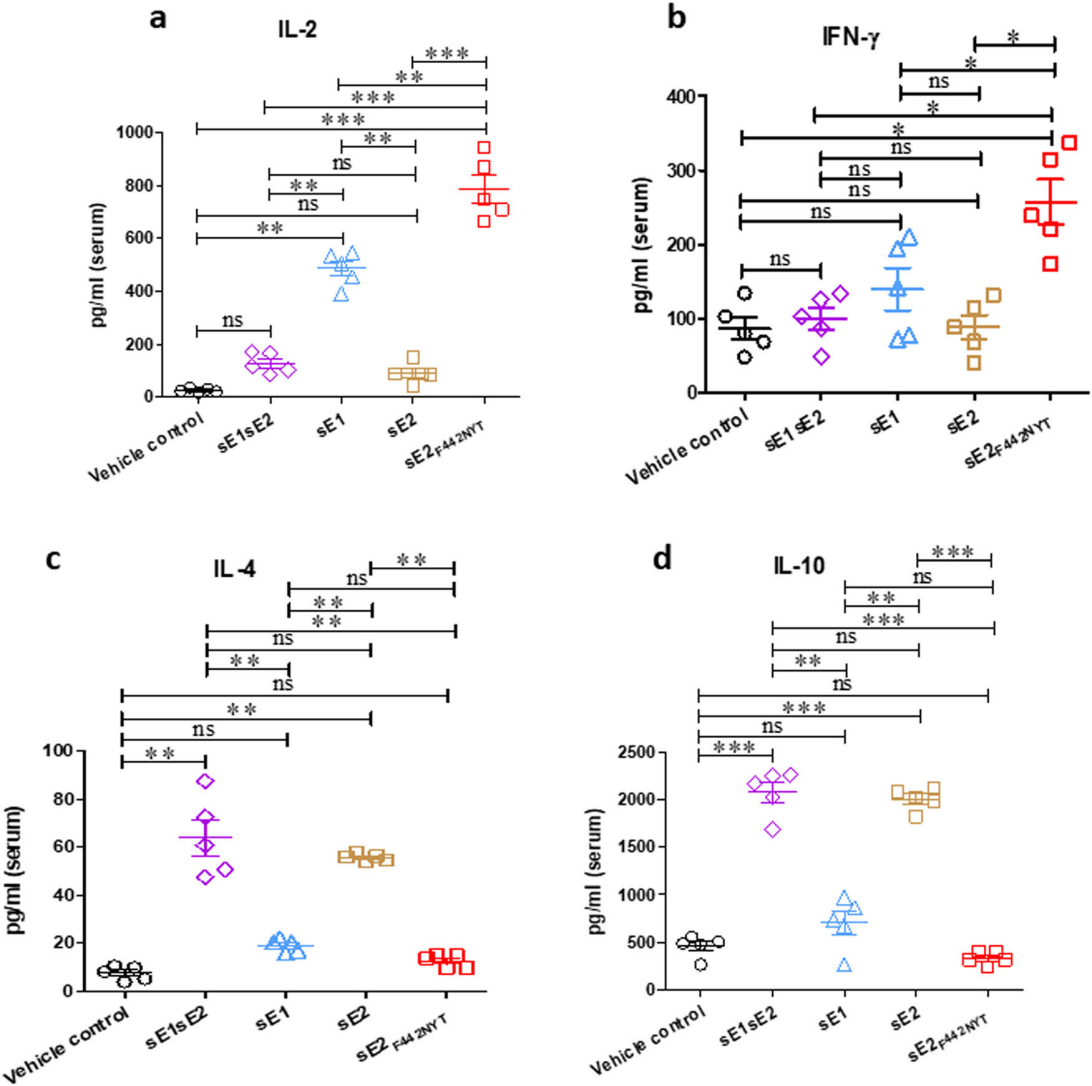
Serum cytokine status of HCV mRNA-LNP vaccinated animals. **a**–**d** The comparative levels of IL-2, IFN-γ, IL-4, and IL-10 were quantified by ELISA from the serum of HCV sE1/sE2, sE1, sE2, or sE2_F442NYT-_ mRNA-LNP immunized mice. The results are presented as the mean with SD. ‘*’, ‘**’, ‘***’ considered statistical significance (t test)with *p*-value of < 0.05, < 0.005, < 0.0005, and ‘ns’ denotes as non-significant.

We further analyzed Th1 and Th2 specific cytokines by ELISA in the serum of HCV envelope-mRNA-LNP vaccinated mice. Th1 specific cytokines, IL-2 and IFN-γ, were significantly elevated in the sE2_F442NYT_ immunized group; whereas, the sE1 alone immunized group showed an enhanced IL-2 and IFN-γ level (Fig. 3a, b). The sE1/sE2 in combination or sE2 alone immunized mice had a low level of Th1 specific cytokines. Conversely, the Th2 specific cytokines, IL-4 and IL-10, were increased in sE1/sE2 in combination or sE2 alone immunized mice. However, the sE2_F442NYT_ or sE1 alone immunized group demonstrated a marginal level of IL-4 and IL-10 (Fig. 3c, d). These results suggest that immunization with sE2 alone induced stronger Th2 specific cytokines while sE1 alone exhibited an opposite effect. Further, combined immunization of sE1/sE2 displayed a cytokine profile like that defined by vaccination with sE2 alone, further reinforcing the modulating nature of sE2. On the other hand, sE2_F442NYT_mRNA-LNP induced Th1 specific cytokines and had a reduced Th2 response in vaccinated mice.

**Fig. 4:**
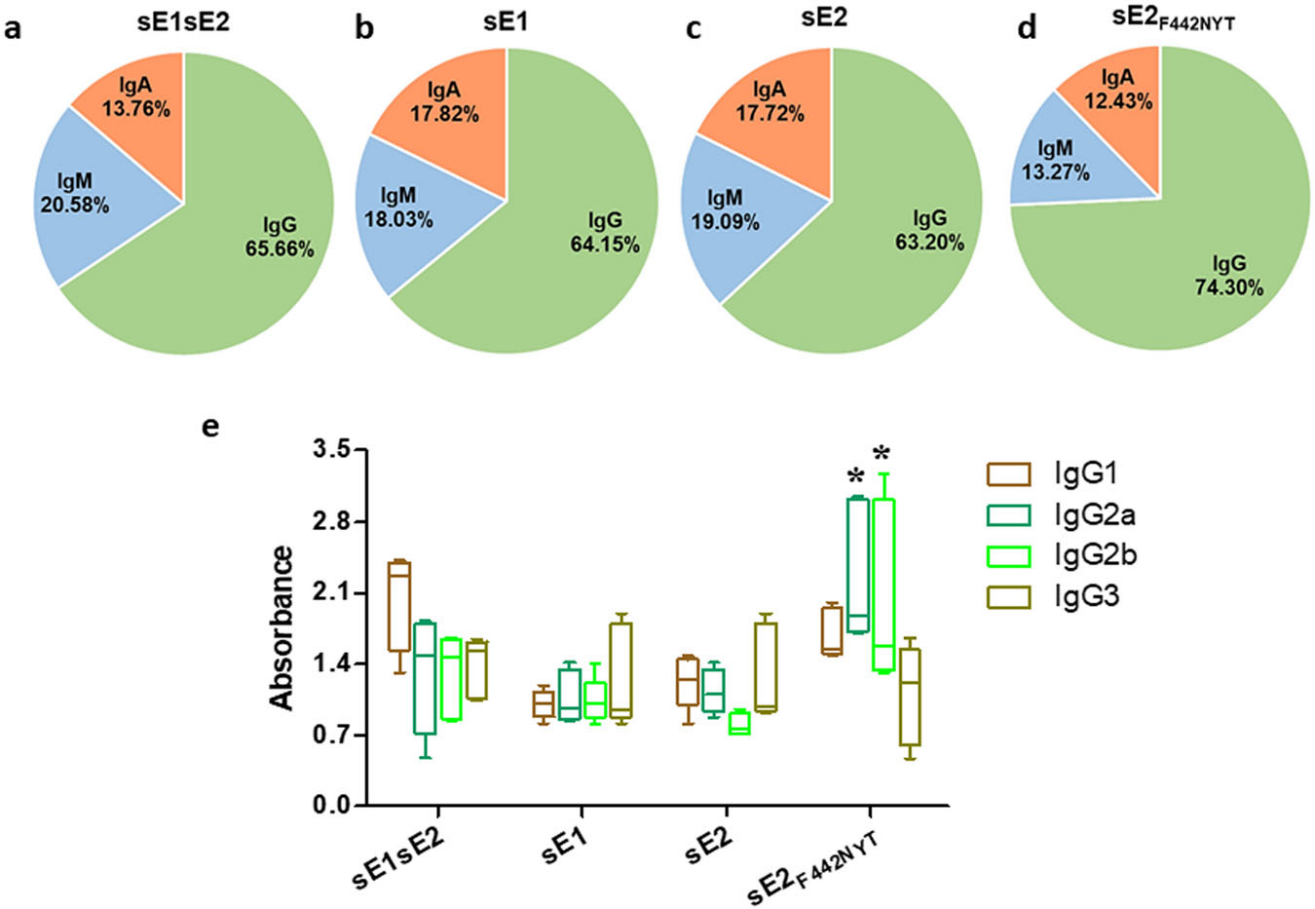
Relative serum immunoglobulins in immunized mice are shown. **a**–**d** Percentage of total IgG, IgM, and IgA were examined from the serum of different antigen immunized mice. **e** Immunized mouse sera were assessed for different IgG isotype (IgG1, IgG2a, IgG2b, and IgG3) specific responses by ELISA. Isotype changes in immunized mice induced by antigens are shown in different color bound boxwith whisker min to max plot. The line inside each box plot shows the median value. The results are presented as the mean with SD, and ‘*’considered statistical significance (ANOVA) with *p*-value of < 0.05.

The distribution of antigen specific immunoglobulin subclasses obtained from the serum of HCV envelope-mRNA-LNP vaccinated animals is shown in pie diagrams (Fig. 4a–d). We observed an increase in total IgG production only in the sE2_F442NYT_ immunized group of mice; whereas the other immunized groups did not show a significant variation in IgG, IgA, or IgM production. We also analyzed IgG specific isotype switching in mouse sera after different HCV envelope antigen exposure. A switch from IgG1 to IgG2a and IgG2b was observed in sE2_F442NYT_ immunized mice, and isotype switching was not observed upon immunization with the other HCV specific mRNA-LNPs (Fig. 4e).

### Inclusion of sE1 with sE2_F442NYT_ may be beneficial for HCV vaccine generation

**Fig. 5:**
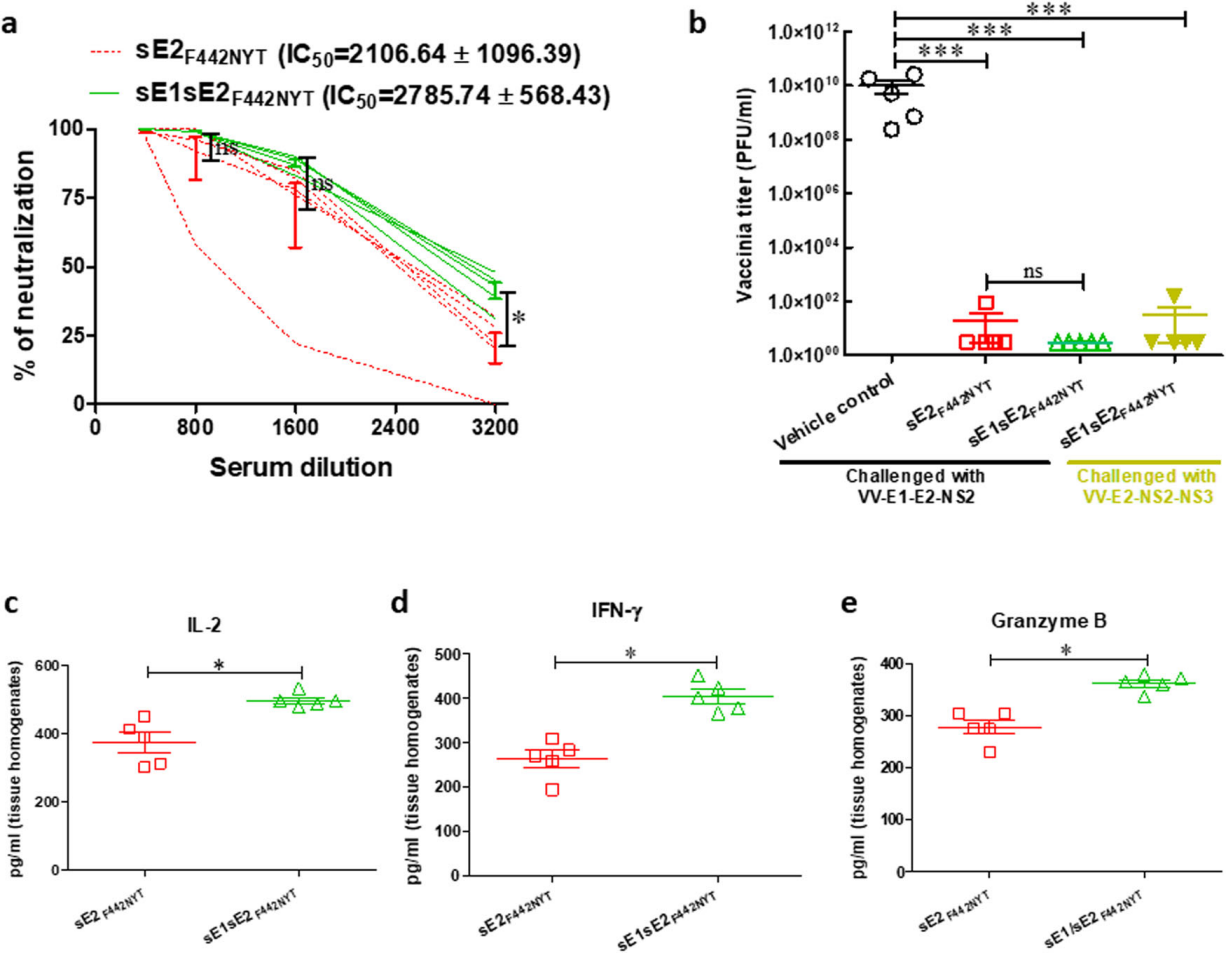
Protection from HCV sE1/sE2_F442NYT_ mRNA-LNP immunization of mice. **a** HCVpp neutralization by mouse sera immunized with sE2_F442NYT_ alone or together with sE1 vaccine antigen. **b** Result from surrogate vv/HCVE1-E2-NS2_134–966_ or vv/HCVE2-NS2-NS3_364–1619_ challenge infection is shown as vaccinia virus recovery by plaque assay from clarified ovary homogenate. **c**–**e** IL-2, IFN-γ, and Granzyme B levels were measured by ELISA from clarified ovary homogenates of immunized mice. The results are presented as the mean with SD. ‘*’, ‘***’ considered statistical significance (Mann-Whitney U, ANOVA and *t*-test) with *p*-value of < 0.05, < 0.0005, and ‘ns’ denotes as non-significant.

We have identified that the use of sE1 as a vaccine antigen has moderate protective efficacy from antibody neutralizing responses, and from the significant induction of CTL-mediated immunity in a mouse model. Next, we examined whether combined immunization of sE1 and sE2_F442NYT_ further increases the protective efficacy of the vaccine preparations. The combined sE1/sE2_F442NYT_ immunization showed ~10–20% enhancement of HCVpp neutralization potency in comparison to sE2_F442NYT_ immunized mouse serum alone (IC_50_ = 2785.74 ± 568.43 Vs. 2106.64 ± 1096.39) (mean ± SD) (Fig. 5a). HCV E1 glycoprotein has distinct pan-genotype neutralizing and T-cell epitopes. Therefore, the addition of sE1 with sE2_F442NYT_ mRNA-LNP could widen the breadth of the vaccine candidate for virus clearance, particularly in the absence of the suppressing nature of sE2.

We have used a surrogate recombinant vaccinia HCV challenge model to examine the biological relevance of the T cell response as a function for protection from infection. In the surrogate challenge model, we observed that four of the five sE2_F442NYT_ immunized mice did not exhibit a detectable recombinant vaccinia virus titer, and we did not recover virus from five combined sE1/sE2_F442NYT_ immunized mice (Fig. 5b). On the other hand, the presence of sE1 with sE2_F442NYT_ as vaccine antigen did not impair the overall protection level when challenged with another recombinant vaccinia virus expressing HCV E2-NS2-NS3 (Fig. 5b). A significant increase in the IL-2, IFN-γ and granzyme B level in the ovary homogenates of combined sE1/sE2_F442NYT_ vaccinated mice was observed when compared to sE2_F442NYT_ mRNA-LNP immunized group (Fig. 5c–e), and seems to indicate a better cellular immune response for the combination of E1 and the E2 mutant relative to the E2 mutant alone. Thus, our results indicated inclusion of sE1 with sE2_F442NYT_ for vaccination may provide protection benefit.

## Discussion

Observations from our study suggest that the modified sE2_F442NYT_ mRNA-LNP as a candidate vaccine induced a strong protective cellular immune response in a BALB/c mouse model. The use of a surrogate recombinant vaccinia virus/HCV challenge model examined the biological relevance of the T cell response, not antibody, as a function for protection. Further, an enhanced neutralizing antibody response was observed using a homologous HCVpp model while examining for immunogenicity of our candidate vaccines. Importantly, a protective immune response generated by sE1 immunization is suppressed in the presence of sE2 introduced as a mRNA-LNP vaccine. The suppressive effect of sE2 may be due to the generation of an enhanced anti-inflammatory cytokine response. Immunization with sE2 alone induced stronger Th2 specific cytokines, while sE1 exhibited a cytokine profile that more closely resembled a Th1 response. However, combined immunization of sE1/sE2 displayed a cytokine profile like that of vaccination with sE2 alone indicating that sE2 could override the response to E1 vaccination. On the other hand, sE2_F442NYT_ mRNA-LNP vaccinated mice strongly induced Th1 specific cytokines with little to no expression of Th2 associated cytokines.

Granzyme B expression was increased in the ovary homogenates from sE2_F442NYT_ immunized mice. However, sE1/sE2 combination or sE2 alone immunized mice exhibited very low granzyme B expression, while immunization with sE1 alone also showed a significant expression of IL-2, IFN-γ and granzyme B. Although, sE2_F442NYT_ mRNA-LNP candidate vaccine induced a stronger binding and neutralizing antibody response, the finding that enhanced T-cell based immune responses to HCV envelope proteins led to protection in our experimental surrogate vv/HCV challenge model is significant, as recovered viral titers were profoundly reduced in response to sE2_F442NYT_ immunization. The importance of an enhanced T-cell based immune response to HCV envelope specific sequences is apparent, and suggests that our modification to the E2 glycoprotein may promote its effective use as a candidate vaccine. The induction of a greater neutralizing antibody response by sE2_F442NYT_ reflects the qualitative nature of humoral immune response induced by immunization and would not have a direct role of surrogate vv/HCV clearance from challenged experimental animals, but could be significant toward an efficacious vaccine regime.

HCV E2 induces IL-10, affects CD8^+^T cell functions, and inhibits granzyme release^19^. In contrast, the sE2_F442NYT_ immunized mouse serum had a significantly higher level of the pro-inflammatory cytokines, IFN-γ and IL-12, and low levels of the anti-inflammatory cytokine (IL-10) and immuno-regulatory cytokineIL-4. sE2_F442NYT_ immunization potentiates a strong neutralization effect on HCVpp (IC50 = 2106.64 ± 1069.39), while sE1 exerts almost similar (IC50 = 1212.06 ± 714.91) neutralization titer as sE2 immunization did (IC50 = 1115.06 ± 223.79). Combined immunization of sE1 and sE2_F442NYT_ had a small advantage in neutralizing potency (IC50 = 2785.74 ± 568.43). Our results also indicated a significant increase in serum IL-2, IFN-γ and ovary granzyme B in recombinant vaccinia virus challenged sE2_F442NYT_ immunized mice for functional make-up of the effector T-cells. Thus, sE2_F442NYT_ appeared to be a superior antigen to sE2. Further, E1 had moderate protective antigenic properties when evaluated alone that were not associated with native sE2. The sE1/sE2_F442NYT_ combination will likely provide a stronger breadth and greater recognition of multiple antigenic sites. An increase in total IgG production was also observed in the sE2_F442NYT_ immunized mice, while other immunized groups did not show a significant variation in IgG, IgA, or IgM level. A switch from IgG1 to IgG2a and IgG2b was observed in sE2_F442NYT_ immunized mice, and isotype switching was not observed upon immunization with other HCV-specific mRNA-LNP candidate vaccines. The IgG isotype switching further supports a strong Th1 response as evident in sE2_F442NYT_ mRNA-LNP vaccinated mice.

Our results indicated that the use of a soluble wild type E2 could restrict immune response from the mRNA-LNP platform and may provide limited benefit as a component of an HCV vaccination strategy. However, the use of a modified sE2_F442NYT_ mRNA-LNP produced a strong immune response in an HCV vaccine, indicating that epitopes within E2 may be highly immunogenic in a vaccine vehicle. The mRNA-LNP vaccine platform would likely offer an excellent opportunity for use in a multi-genotype HCV vaccine to accommodate many HCV E2 sequences to induce a broad protective immune response, if cross genotype protection proved to be inefficient. The use of both E1 and sE2_F442NYT_ mutant will likely offer a much broader epitope specific repertoire of antibodies and T-cell mediated immune responses, allowing for greater vaccine efficacy despite the potential loss of limited antigenic sites/epitopes from the E2_F442NYT_ modification. There are suggestions for the need of conformationally correct E1 and E2 envelope glycoproteins in HCV vaccines. At this time, we are not sure what optimum conformation and how many neutralizing antigenic sites need to be present for an effective E2 inclusive vaccine. Further, the abundance and frequency of the antibodies to these conformational domains in spontaneously clearing humans appear to remain unknown. Our results suggest that sE1 vaccination induced higher neutralization titers as compared to sE2 or sE1/sE2, and could also induce a T-cell associated cytokine response. Protection against challenge infection in a mouse model may likely be contributed by both CD4^+^ and CD8^+^T cell responses as suggested earlier^20^. Our previous study revealed that HCV E2 binding to CD81 tilts immune function towards a Th2 cytokine associated response. This was evident using a mRNA-LNP vaccine platform that normally promotes Th1-biased immune responses^21,22^ where the presence of E2 led to a shift to Th2 response.

The lack of an immuno-competent small animal model susceptible to HCV infection is a major limitation to our study. The vaccinia virus challenge model is a surrogate system, not reflective of natural HCV infection, and the model only assesses T cell associated protection and gives no indication of the protective role of neutralizing antibodies. Further, our study did not consider in depth the protection for heterologous genotype or pan-genotype-related circulating HCV infection in the communities of different geographic regions. Our ongoing studies focus upon extending our findings to determine the nature of protective immune responses for virus clearance in other animal models, including in nonhuman primates. The role of the antibodies in virus clearance, and the role of memory B- and T-cell responses are the other important aspects to understand from in vivo studies. The ability of any vaccine candidate to cross-neutralize multiple HCV genotypes must also be fully explored.

Immunization with the sE2_F442NYT_ mRNA-LNP induces a much improved proinflammatory cytokine response, T-cell response, immunoglobulin class switching, neutralizing antibody induction, and a strong T-cell based protective efficacy against a surrogate vv/HCV challenge infection in mice. Our study limitation also includes immunization and infection of only female mice as a model and challenge with a HCV surrogate virus due to constraints with the availability of small animals permitting HCV infection for candidate vaccine evaluation. To enhance the protective efficacy of the variable HCV E2, we observed that the use of a relatively conserved E1 ectodomain alone also induces a moderate protective T-cell response. Booster immunization with peptides representing conserved B-cell and T-cell epitopes from multiple HCV genotypes to mRNA-LNP vaccinated mice may also find additional benefits. Thus, our results open new avenues for considerable advancements of HCV vaccine development by incorporating sE1/sE2_F442NYT_ in an mRNA-LNP platform for induction of a broad protective immune response. Our observations are important and hold a strong potential for understanding the immunogenicity of selected antigens for a candidate vaccine in higher primates and humans to accelerate HCV vaccine development.

## Methods

### Generation of mRNA-LNP vaccines encoding HCV envelope glycoproteins

Codon optimized luciferase, and HCV (genotype 1a/H77C) envelope glycoproteins sE1 (aa 193–351), sE2 (aa 386–660), and modified sE2_F442NYT_ specific sequence were constructed. HCV sE2 and sE2_F442NYT_ protein samples were examined and compared for the inserted glycosylation site at F442 after PNGaseF treatment at the Washington University Proteomics Shared Resource (WU-PSR), St. Louis. The glycosylation site at the residue F442 of modified sE2_F442NYT_ protein was observed following constitutive deamidation of that residue and variable deamidation of other asparagine residues in that sequence. The mRNAs from HCV sE1, sE2, or sE2_F442NYT_ were generated, purified, and encapsulated into LNPs for use as candidate vaccines in this study^8^.

Codon-optimized luciferase HCV E1_193–351_ and HCV E2_386–660_ specific sequences with or without mutations at residues 442 and 444 were synthesized into mRNAs, purified and encapsulated into LNPsfor use in mouse immunization. The ethanolic lipid mixture comprising ionizable cationic lipid, phosphatidylcholine, cholesterol, and polyethylene glycol-lipid was rapidly mixed with an aqueous solution containing cellulose-purified N1-mΨ in vitro-transcribed mRNAs. The mRNA-loaded LNPs were formulated using a total lipid concentration of 40 mM. RNA-loaded particles were characterized by size, surface charge, encapsulation efficiency, and endotoxin content and were stored at −80 °C at an RNA concentration of 1 μg/μL (in the case of loaded particles) and a total lipid concentration of 30 μg/μL (both loaded and empty particles). The mean hydrodynamic diameter of mRNA-LNPs was ~80 nm, with a polydispersity index of 0.02 to 0.06 and an encapsulation efficiency of ~95%. The LNP formulation used in this study is proprietary to Acuitas Therapeutics (US patent 10,221,127). Single use aliquots of the vaccine preparation were used for mouse immunization.

### Immunization of mice with mRNA-LNP vaccine and challenge infection using recombinant vaccinia virus

BALB/c mice (Jackson Lab) were divided into five groups (5 mice per group) and each group of mice were immunized intra-muscularly with 10 μg mRNA-LNP candidate vaccine as sE1/sE2, sE1, sE2, sE2_F442NYT,_ sE1/sE2_F442NYT,_ or vehicle control twice at 2-week intervals. HCV vaccinia challenge as a surrogate model is helpful in analyzing protective response to challenge infection^8,20,23,24^. Test bleeds (3 days before immunization and 3 days before challenge infection) from mice were analyzed. The use of the surrogate recombinant vaccinia virus(vv)/HCV challenge model in our study examined the biological relevance of T cell response, not the antibody, as a function for protection from challenge infection.

Immunized mice were challenged intra-peritoneally with live recombinant vaccinia virus expressing HCV E1-E2-NS2_134–966_ or HCVE2-NS2-NS3_364–1619_ (genotype1a/H77C), and sacrificed 4 days after challenge infection for collection of the ovaries for further analysis. Ovaries were homogenized, freeze-thawed three times and centrifuged. Clear supernatant was serially diluted for measuring vaccinia virus titer by plaque forming assay on BSC-40 cell monolayer. After 3 days, plaques were stained with 1% crystal violet and counted.

### Ethical statement

All animal experiments were conducted in accordance with the relevant local, state, and federal regulations. The studies were approved by the Saint Louis University Institutional Animal Care and Use Committee (IACUC).

### Cytokine quantification

Test bleeds (3 days before immunization and 3 days before challenge infection) from experimental mice were analyzed. The serum cytokines, IL-2 (Sigma, RAB0287), IFN-γ (R&D Systems, DY485), IL-4 (Biolegend, 431101), and IL-10 (Invitrogen, 88–7105–22) were measured from the immunized mice by ELISA using commercially available kits following the manufacturer’s instruction and dilution guidelines. Similarly, mouse IL-2 (Sigma, RAB0287), IFN-γ (R&D Systems, DY485) and Granzyme B (R&D Systems, DY1865) were quantified from ovary homogenates of the mice after challenge infection with recombinant vaccinia virus.

### HCV pseudoparticle neutralization assay

HCV pseudo particles (HCVpp) were generated by co-transfection of HEK293T cells with the HDM-Hgpm2-pRC-CMV-Rev1b-HDM-tat1b packaging vector, Luciferase-IRES-ZsGreen plasmid (BEI Resources), and plasmids expressing E1E2 from HCV genotypes 1a (H77C), 1b (1b58), 3 (3.1.2), and 4(4.1.2) available in our laboratory or obtained from Justin Bailey (Johns Hopkins University School of Medicine, MD, USA) using Lipofectamine 3000 (Invitrogen L3000–008)^23^. Supernatants containing HCVpp were harvested 72 h post-transfection and filtered through a 0.45 μm pore size nitrocellulose membrane. For neutralization testing of HCVpp, 1.5 × 10^4^ Huh7.5 cells per well were plated in 24-well tissue culture plate and incubated overnight at 37 °C. The following day, HCVpp were mixed with or without different serial dilutions (1:100, 1:200, 1:400, 1:800, 1:1600, 1:2400, 1:3200) of the immunized mouse sera and incubated for 1 h at 37 °C before adding to Huh7.5 cells. After 72 h at 37 °C, cells were lysed with cell lysis buffer (EI53A, Promega) and 100 μl of luciferase substrate (EI51A, Promega) was added to each well. Luciferase activity was measured in relative luminescence units (RLU) using Glomax luminometer (Promega). The inhibitory concentration of neutralization of pseudotype infectivity (IC50) was defined as ≥ 50% reduction of luciferase activity using the following formula^6^. The percentage of neutralization was calculated as [1 − (RLU_mAb_/RLU_untreated_)] × 100, with the untreated control RLU values averaged from triplicate. Neutralizing activities were presented with dilutions of the serum samples using lower and upper bounds (0% and 100% inhibition).

### Subclass or isotype specific immunoglobulin response

Nunc MaxiSorp ELISA plates were coated with 1 µg/ml purified HCV E1E2 proteins (Chiron) in 50 µl 0.1 M sodium bicarbonate buffer (pH 7.2) overnight at 4 °C. The wells were blocked with 2.5% BSA/PBS blocking buffer for 2 h at 37 °C and washed four times with 0.01% Tween 20 in PBS. Mouse sera were serially diluted (1:50, 1:100, 1:200) in blocking buffer, added to the plate, and incubated overnight at 4 °C, followed by washing. Different rabbit HRP-tagged anti-mouse immunoglobulins (subclass: IgG, IgM, IgA; and isotype: IgG1, IgG2a, IgG2b, IgG3) (Bio-Rad, Mouse Typer Isotyping Panel, 1722055) were added to the appropriate wells and incubated for 1 h at 37 °C, followed by four washes. HRP conjugate was added at 1:3000 and incubated for 1 h, and the wash cycle was repeated. Next, 100 μl of peroxidase substrate solution was added and the reaction was stopped with 2 M sulfuric acid. The absorbance was measured at 450 nm using an ELISA plate reader (Tecan).

### HCV envelop peptide specific reactivity

Peptide-specific serum IgG binding was tested using ELISA with 18-mer peptides from E1 (aa 314–331), E2 (aa 404–421) and E2 (aa 429–446) of HCV H77 strain (NR4062 and NR4063, BEI Resources) representing diverse genotype specific conserved neutralizing epitopes^9,10,12–15^. A SARS-CoV-2 fusion domain peptide was used as an irrelevant control. Wells were coated with 500 ng of peptide and ELISA was performed using different dilutions (1:200, 1:400, 1:800) of immunized mouse sera as described above.

### Statistical analysis

All data were analyzed using GraphPad Prism 7 software. Analysis of data with only two groups was conducted utilizing a one-tailed Mann-Whitney U test or unpaired *t*-test. One-way ANOVA with Kruskal-Wallis’s test was used for multiple pairwise comparisons between groups. All the data are expressed as Mean ± SD (standard deviation of the mean), and *p* < 0.05 was considered statistically significant.

### Reporting summary

Further information on research design is available in the Nature Research Reporting Summary linked to this article.

## Supplementary information

Reporting Summary

## Data Availability

Data associated with this study are presented in this paper.
